# Vitamins and Mineral Supplements for Retinitis Pigmentosa

**DOI:** 10.1155/2019/8524607

**Published:** 2019-02-21

**Authors:** Ying Zhao, Kai Feng, Ruibao Liu, Jinhua Pan, Lailin Zhang, Xuejing Lu

**Affiliations:** ^1^Chengdu University of Traditional Chinese Medicine, 37 Twelve Bridge Road, Chengdu 610075, China; ^2^Beijing University of Chinese Medicine, 11 East North Three Huan Road, Beijing 100029, China

## Abstract

Retinitis pigmentosa (RP) is a group of inherited progressive retinal dystrophies that is present with progressive vision loss, night blindness, visual field reduction, and retinal pigmentation of the fundus. RP is an uncommon but clinically important disease. It is progressive and potentially blinding, and to date, no cure for RP has been identified and clinical interventions to retard disease progression are limited. Because of the nature of this disease, there has been great interest in the development of therapeutic interventions that may prevent its progression or restore the loss of visual function. Studies have indicated a possible role of vitamins and minerals in preventing the progression of RP: vitamin A has been reported to have an important role in the function of retinal photoreceptors; lutein is assumed to play a preventive role in fundus diseases; and docosahexaenoic acid, which is found within photoreceptor cell membranes, may have a functional role in preventing the progression of RP. Therefore, this study aimed to systematically review data from randomized clinical trials (RCTs) evaluating the safety and efficacy of vitamins and mineral supplements for the treatment of RP. We searched through relevant trials in the Cochrane Library, PubMed, Embase, Ovid, AMED, OpenGrey, ISRCTN registry, http://ClinicalTrials.gov, and the WHO ICTRP ranging from the respective dates of foundation to June 18, 2018. We reviewed eight randomized control trials (RCTs) with data for 1231 patients. The results indicated that patients with RP may experience delayed disease progression with vitamin and mineral supplementation. In a broader sense, this review suggests that the future trials on RP patients should consider more vitamins or mineral supplements and other outcome measures from the trials included in this review.

## 1. Background

Retinitis pigmentosa (RP) is a group of hereditary retinal diseases characterized by progressive degeneration of rod and cone photoreceptors. Patients often develop loss or impairment of night vision during puberty, peripheral vision loss appearing in the form of tubular vision, and even central vision loss in the late stage due to degeneration of rod cells and cone cells along with the progressive loss of function. Globally, the incidence of RP is 1 : 4000 to 1 : 5000, making it the most common blinding genetic eye disease [1, 2]. RP includes nonsyndromic retinitis pigmentosa (NSRP) and syndromic RP (SRP). The clinical manifestations of NSRP patients include ocular abnormalities, and NSRP can be divided as follows according to the genetic characteristics: autosomal dominant retinitis pigmentosa (ADRP), autosomal recessive retinitis pigmentosa (ARRP), and X-linked retinitis pigmentosa (XLRP) [3]. SRP presents in about 20% to 30% of the patients, and there are about 30 different types of SRP, of which the more common types are Usher syndrome and Bardet–Biedl syndrome [4]. The clinical manifestations of RP include decreased vision, night blindness, progressive visual field defects, ocular findings including waxy pallor of the optic discs, attenuated retinal vessels, and intraretinal pigment around the midperiphery. The majority of the patients develop central posterior subcapsular cataracts. Patients show elevated final dark adaptation thresholds with reduced and delayed electroretinography (ERG) findings.

The mechanism underlying cone cell death in RP is uncertain. Rods cells are the key consumers of oxygen in the retina and, after the rods cells die, the level of oxygen in the outer retina increases. Photoreceptors in the retina are subject to oxidative stress throughout life due to combined exposures to light and oxygen. Oxidative damage has been proposed to be an important contributor to cone cell death in RP, and antioxidants may prevent cone cell damage in RP by reacting with free radicals produced in the process of light absorption [5, 6]. It is likely that the benefits will be additive or synergistic by reducing oxidative damage to cones while simultaneously increasing the threshold for apoptosis with neurotrophic factors. Any vitamin or mineral that is known to have antioxidant properties in vivo or is an important component of an antioxidant enzyme present in the retina is deemed to be therapeutic for RP. Some researchers have undertaken studies on vitamins and nutritional supplements, hoping to improve patients' functional vision or at least slow down the course of RP. Studies have indicated a possible role for vitamins or minerals in preventing the progression of RP, and data from the trials generally support a link between nutritional factors, particularly those with vitamin A, and the risks of RP [7–10]. Vitamin E is well known as an antioxidant that protects the structure of photoreceptors in vitro and in vivo [11, 12]. Lutein, a carotenoid, is assumed to play a preventive role in fundus diseases [13–16] and has been deemed to be a possible therapeutic agent that can help in preserving the visual function of patients with RP. Therefore, we speculated that vitamins and mineral supplements might represent one of the candidates that can avert photoreceptor apoptosis, thus preventing further development of RP.

## 2. Methods

### 2.1. Criteria for considering Studies for This Review

#### 2.1.1. Types of Studies

We included randomized controlled trials (RCTs) comparing vitamins or mineral supplementations (alone or in combination) to placebos or no intervention in RP patients. We excluded RCTs currently recruiting participants on the clinical trial registration platform (http://www.clinicaltrials.gov) (http://www.who.int/ictrp/search/en) (http://www.isrctn.com/) because there are no clinical data available for us to use.

#### 2.1.2. Types of Participants

Participants in the included trials were individuals with a confirmed diagnosis of RP.

#### 2.1.3. Types of Interventions

We included vitamins or mineral supplements that are known to have antioxidant properties in vivo or have been shown to be beneficial for retinal diseases, such as vitamin A, vitamin C, vitamin B, vitamin E, vitamin B12, beta-carotene or *β*-carotene, carotene, cobalamin, antioxidants, carotenoid, zinc, riboflavin, selenium, lutein, xanthophylls, zeaxanthin, micronutrient, provitamins, multivitamins, fish oils, trace elements, and trace minerals.

#### 2.1.4. Types of Outcome Measures

The primary outcome selected for this review was mean change in the visual field assessed with the Humphrey field analyzer or Goldmann perimeter. Secondary outcomes included the log-mean change in ERG amplitude and the change in logMAR visual acuity.

### 2.2. Search Methods for Identification of Studies

We searched through and looked up the related literature in the Cochrane Library, PubMed, Embase, Ovid, AMED (Allied and Complementary Medicine Database), OpenGrey (System for Information on Grey Literature in Europe) (http://www.opengrey.eu/), the ISRCTN registry (http://www.isrctn.com/), http://ClinicalTrials.gov (http://www.clinicaltrials.gov), and the WHO International Clinical Trials Registry Platform (ICTRP) (http://www.who.int/ictrp/search/en) from the respective foundation dates to June 18, 2018. We did not use any language restrictions in the electronic search for the trials. Two researchers independently extracted data from the publications and evaluated the risk of bias for all the included trials. We also contacted the investigators for further research information or for trials with unpublished results. Then, we cross-checked the literature and asked for assistance and guidance from the relevant experts whenever there was a difference in opinions.

### 2.3. Assessment of Risk of Bias in Included Studies

We used the risk of bias assessment tool recommended by the Cochrane Handbook 5.3, which included six items: random sequence generation, allocation concealment, blinding of subjects and intervention providers (blinding of participants and caregivers), blinding of outcome assessments, incomplete outcome data, selective reporting, and other biases. The criteria for assessing the risk of bias were low risk, high risk, and unclear.

### 2.4. Measures of Treatment Effect

The odds ratio (OR) was used as the count data, and the standard mean difference (SMD) was used as the measurement data. Both of these used 95% confidence interval as the effect amount. We assessed heterogeneity by the Cochrane *I*^2^ test. If *I*^2^ < 30%, the heterogeneity was small; if 30% < *I*^2^ < 50%, the heterogeneity was moderate. We planned to use a fixed-effect model; *I*^2^ > 50% indicated a high degree of heterogeneity among the results. We first analyzed the source of heterogeneity and performed a subgroup analysis; if there was no source of heterogeneity, we performed a descriptive analysis.

### 2.5. Adverse Effects

We listed all adverse consequences reported in the included studies. The adverse events included systemic complications such as liver and kidney function damage and severe blood disease.

## 3. Results

### 3.1. Description of Studies

The characteristics of the included studies and the excluded studies are represented in Tables 1 and 2, respectively.

### 3.2. Literature Search Results

**Figure 1:**
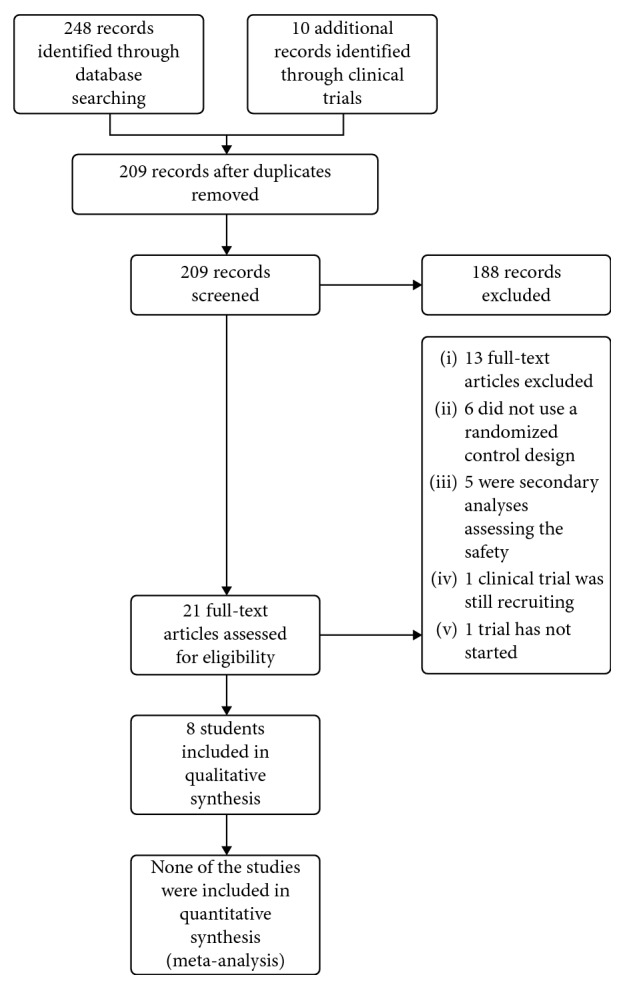
Flowchart depicting the selection of studies included in the review.

We retrieved a total of 248 titles and abstracts and 10 http://ClinicalTrials.gov records during the electronic searches. After removing 49 duplicate records, we reviewed 209 titles and abstract records for eligibility and excluded 188 of these records. Two researchers studied and looked up the remaining 21 related records and reviewed the full text. We excluded 13 records for the following reasons: six did not use a randomized control design; five were secondary analyses assessing the safety; one clinical trial was still recruiting; and the recruitment for one trial had not started. The flowchart describing the literature search is shown in Figure 1.

### 3.3. Types of Participants

The participants of the eight selected studies included patients from USA, Canada, Mexico, or Israel. Six studies recruited both men and women [17–19, 21–23]; two studies recruited only men [20, 24]. A total of 1335 participants were enrolled, and 1231 were analyzed in the included studies for this review. All participants had a confirmed diagnosis of typical RP. Three studies described the genetic type of the participants' RP [17, 19, 22]. Three studies' clinical diagnosis was consistent with X-linked inheritance [20, 24]. The recruitment age ranged from 10 to 55 years, and one study included children and younger participants (4–38 years) in comparison with the other trials [20]. Seven studies elaborated the sample size estimation, except Pasantes-Morales et al. [18] and Rotenstreich et al. [23]. Refer to Table 1 for further details.

### 3.4. Types of Interventions

The review included eight trials that evaluated the effects of single vitamins on RP and also evaluated the effects of combination vitamins on RP. In the trial by Berson et al. [17], the groups received different doses of vitamin A (15000 IU/d, 75 IU/d) plus vitamin E 3 IU/d or vitamin E 400 IU/d. Vitamin A was administered as retinyl palmitate and vitamin E as di-a-tocopherol in soft gelatin capsules. The treatment duration was 4 years. In the trial by Pasantes-Morales et al. [18], the groups received diltiazem 30 mg and vitamin E 400 mg or taurine 1 g. The daily treatment included taurine 1 g, vitamin E 400 mg, and diltiazem 30 mg. The treatment duration was 3 years, but 19 patients in the treatment group received treatment for longer than 3 years (3–6 years). In the study by Hoffman et al. [20], the groups received docosahexaenoic acid (DHA) 400 mg/d or placebo. The treatment duration was 4 years. In another study by Berson et al. [19], the groups received DHA 1200 mg/d plus vitamin A 15000 IU/d or vitamin A 15000 IU/d. Vitamin A was administered as retinyl palmitate. The treatment duration was 4 years. In the study by Bahrami et al. [21], the groups received lutein capsules 10 mg/day for 12 weeks, 30 mg/day for 12 weeks, or placebo for 24 weeks, and all participants were offered multivitamin supplementation. In the third study by Berson et al. [22], the groups received lutein 12 mg plus vitamin A 15000 IU/d or a control tablet plus vitamin A 15000 IU/d. The treatment duration was 4 years. In the trial by Rotenstreich et al. [23], the groups received capsules containing 300 mg of the 9-cis *β*-carotene-rich alga *Dunaliella bardawil* or placebo (starch) 300 mg. The treatment duration was 90 days. In another trial by Hoffman [24], the groups received 200 mg algal-derived DHA or corn/soy (placebo). Each patient was provided a sufficient number of capsules to achieve a dose of 30 mg DHA/kg/d. The total DHA dosage ranged from 600 to 3600 mg/d. Treatment duration was 4 years. Refer to Table 1 for further details.

### 3.5. Types of Outcome Measures

The primary outcome was the visual field values, whereas the secondary outcomes were visual acuity and ERG findings, which were measured annually [17, 19, 22]. Similarly, in the study by Pasantes-Morales et al. [18], the primary outcome was the visual field values, whereas the secondary outcomes were visual acuity and ERG findings, which were obtained every 4 months. In the study by Hoffman et al. [20], the primary outcome was cone ERG measurements (31 Hz electroretinogram amplitude), while the secondary outcomes were visual field values, visual acuity values, and the results of blood and fatty acid analysis, which were performed annually. In the study by Bahrami et al. [21], the primary outcome was visual acuity, while the secondary outcomes were contrast sensitivity and central visual field radius, which were measured every 24 weeks. In the study by Rotenstreich et al. [23], the primary outcome was the dark-adapted ERG maximal b-wave response amplitude, whereas the secondary outcomes were visual acuity and light-adapted ERG b-wave response amplitudes, dark-adapted chromatic visual field area, and conventional light-adapted visual field area, which were measured every 90 days. In another study by Hoffman et al. [24], the primary outcome was the cone ERG (31 Hz electroretinogram amplitude) findings, while the secondary outcomes were rod ERG amplitude and maximal ERG amplitude, which were measured annually. Our primary outcome, visual field sensitivity, was reported as the secondary outcome in two studies by Hoffman et al. [20] and the study by Rotenstreich et al. [23]. ERG findings as the primary outcome were measured in the two studies by Hoffman et al. [20] and Rotenstreich et al. [23]. Our secondary outcome, visual acuity, was measured as the primary outcome in the study by Bahrami et al. [21].

### 3.6. Risk of Bias in the Included Studies

**Figure 2:**
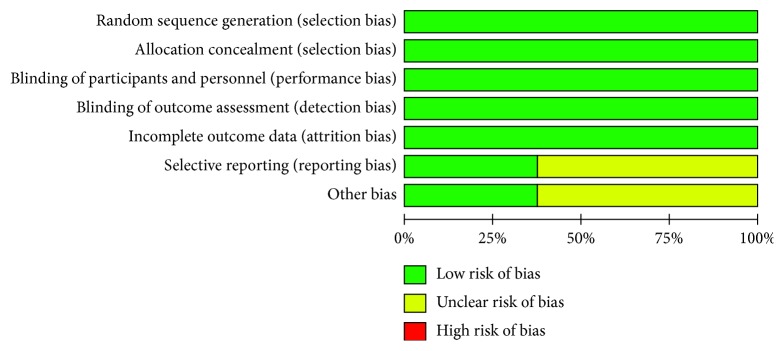
Risk of bias assessment: graph depicting review authors' judgements about each risk of bias element presented as percentages across all the included studies.

**Figure 3:**
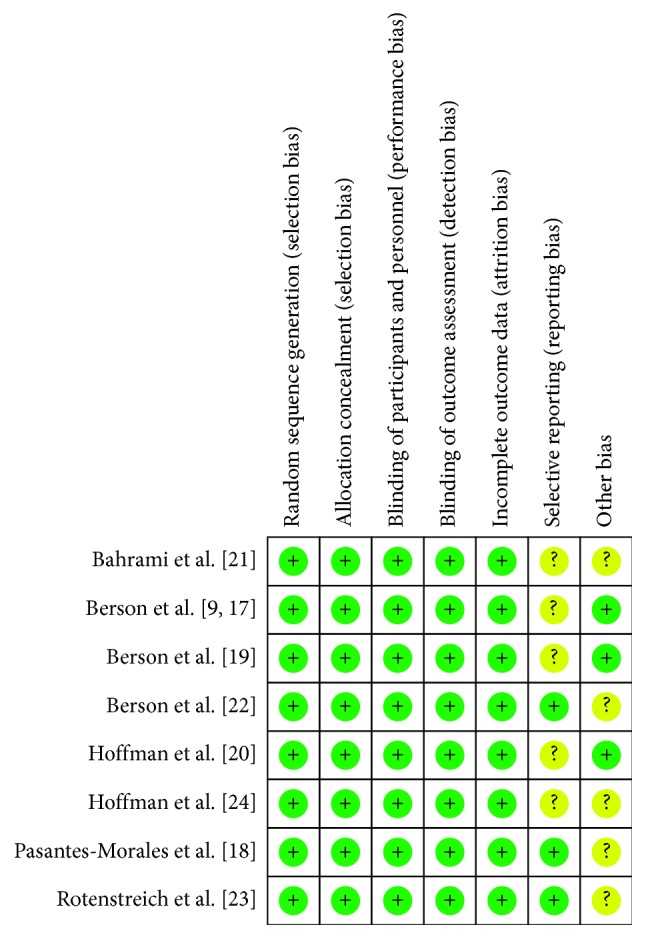
Risk of bias summary: review authors' judgements about each risk of bias element for each included study.

We assessed the risk of bias for all eight included trials using the six domains described in the risk of bias assessment tool. Blinding of outcome and incomplete data were divided into three criteria based on primary and secondary outcomes, so a total of 12 criteria have been listed in the table (Table 1). The summary of the risk of bias assessment is shown in Figures 2 and 3.

### 3.7. Visual Field

Three trials reported the treatment effects associated with DHA [19, 20, 24]; two trials, with lutein [21, 22]; one trial, with vitamin A [17]; one trial, with vitamin E [18]; and one trial, with 9-cis *β*-carotene-rich alga [23]. Out of these, four trials reported the visual field values as the primary outcome [17–19, 22], while two trials reported these values as the secondary outcome [20, 21].

As for the four trials that reported the visual field values as the primary outcome, we found no significant effect of vitamin A or vitamin E in one of the trials analyzed for field area [17]. A protective effect of taurine and vitamin E on visual field loss was observed in the study by Pasantes-Morales et al. [18], which reported a reduction in the visual field loss in the treated group and that the magnitude of the decrease in the central area was more obvious in the control group −4.05 (SE ± 0.70) than in the treated group −2.59 (SE ± 0.98). No significant difference in the decline of visual field sensitivity was noted between the DHA plus vitamin A and the control plus vitamin A groups [19]. No intergroup differences were found in the visual field values in study by Hoffman et al. [20]; this study suggested that DHA intervention alone does not protect against visual field damage. On the other hand, lutein treatment showed a statistically significant effect on visual field (*P*=0.038) [21], and the effect was enhanced in the model assuming a 6-week delay in activation of lutein after the intake. Another trial showed no significant difference in the primary outcome of central visual field sensitivity with the 30-2 program between the lutein and control groups, but it showed a significant difference in the secondary outcome of midperipheral visual field sensitivity with the 60-4 program (*P*=0.05) [22]. It also showed that patients with the highest serum lutein level or with the highest increase in macular pigment optical density was slower in 60-4 program (*P*=0.01 and *P*=0.006, respectively) and also had the slowest decline in HFA 30-2 and 60-4 combined field sensitivity (*P*=0.005). This indicated that lutein supplementation of 12 mg/d slows down the loss of midperipheral visual field on an average among the nonsmoking adults with retinitis pigmentosa taking vitamin A. No significant intergroup differences were observed for the visual field values upon 9-cis *β*-carotene intake in the study by Rotenstreich et al. [23]. However, treatment with 9-cis *β*-carotene significantly increased retinal function in RP patients in dark-adapted maximal b-wave and light-adapted b-wave. Another study by Hoffman et al. [24] reported that the annual visual field sensitivity progression for the foveal, macular, peripheral, total, and ellipsoid zone regions were reduced by DHA supplementation (*P*=0.039, *P*=0.031, *P* < 0.0001, *P* < 0.0001, and *P*=0.033, respectively).

### 3.8. Visual Acuity

A total of seven trials reported visual acuity as a secondary outcome indicator. No significant difference was found in ETDRS visual acuity with different doses of vitamin A and vitamin E in RP [17]. Visual acuity was comparably preserved among all the treatment groups, and there were no data to predict a beneficial effect of vitamin A on the decline of visual acuity over a long term. However, in one study, visual acuity increased by 24% of the control and 40% of the taurine and vitamin E treated patients [18]. And, the long-term benefits of the taurine/vitamin E treatment were evident in the group receiving the formulation for up to 6 years. No significant intergroup differences in ETDRS visual acuity were observed in another study [19], which showed that 1200 mg/d DHA was not substantial in improving the visual acuity of RP patients. In the study by Hoffman, oral administration of 400 mg/d of DHA did not improve the visual acuity; mean difference in visual acuity was 0.06 + 0.2 (logMAR) in the placebo group and 0.05 + 0.23 (logMAR) in the DHA group (*t* = 0.15; *P*=0.88) [20]. Similarly, there was no statistically significant difference in visual acuity in the studies by Bahrami et al. [21] (*P*=0.89) and Berson et al. [22] (*P*=0.80). Compared to the progress of vision loss against the natural loss expected to occur over the course of 48 weeks, lutein supplementation might slightly improve the visual acuity [21]. No significant intergroup differences were found in the best-corrected visual acuity with 9-cis *β*-carotene supplements in RP (*P*=0.90) [23].

### 3.9. ERG

As for the decline in the 30-Hz ERG amplitude, Berson et al. [17] concluded that the patients receiving vitamin A 15000 IU/d displayed a slow decline than those not receiving this dosage (*P* < 0.001). In addition, patients receiving vitamin A 15000 IU/d plus vitamin E 3 IU/d exhibited a significantly slower decrease than the trace group (*P*=0.03). However, use of high-dose supplements of vitamin E, such as 400 IU/d, could lead to a decrease in serum retinol concentration, which may affect the course of retinal degeneration in RP. In another study, analysis of ERGs showed that the mean annual decline of the remaining retinal function was 9.92% in the DHA plus vitamin A group, lower than the plus vitamin A group (10.49%), but there was no statistically significant difference (*P*=0.64) [19]. It has also been observed that the average loss of cone amplitude in the DHA group was 25% less than that in the placebo group (mean ± SD; −0.199 ± 0.172 vs. −0.266 ± 0.173; log_*μ*_ V; *P*=0.20) [20]. A daily oral DHA administration throughout the 4-year trial reported no group differences in rod ERG and maximal ERG (*P*=0.27; *P*=0.65) [24]. No effect of lutein was detected with respect to preserving the full-field 30 Hz cone ERG (*P*=0.59) [22]. Furthermore, 300 mg/d of 9-cis *β*-carotene treatment showed an improvement in objective rod-cone functions significantly, as reflected by the changes in maximal dark-adapted ERG b-wave amplitude responses [23].

### 3.10. Adverse Effects

Four studies reported adverse effects after the follow-up year. Berson [17] evaluated the effect of the vitamin A plus vitamin E treatment that resulted in a worsening condition only in one patient. According to Bahrami et al. [21], one participant receiving lutein and two participants receiving the placebo showed impaired liver function test results during the 6-week visit, but in all three, the serum liver enzyme levels returned to normal when the tests were repeated. At the start of the study, two participants ceased multivitamin ingestion due to a serious intolerance to multivitamin (“stomach upset”). Berson et al. [22] reported one patient in the control plus vitamin A group who died in a motorcycle accident after year 3 of follow-up. Two patients in the lutein plus vitamin A group showed slight elevation in serum liver enzyme levels of unknown etiology at year 4. However, there was no evidence of these effects attributable to the vitamin A or lutein based on the assessments of blood and serum liver function. Hoffman et al. [24] reported one participant with a family history of Crohn's disease who was sensitive to DHA supplementation.

## 4. Discussion

This review provides evidence showing that vitamin supplements do affect retinal function in different ways. The criteria we originally selected were the effects of vitamins and minerals on retinitis pigmentosa. However, due to the limitations of existing literature, only eight published RCTs were retrieved. In these RCTs, only a small number of vitamins were reported to have an effect on retinitis pigmentosa, such as vitamin A or lutein or DHA. Vitamin A has always been a trace element of interest for nutritionists. It is not a single nutrient but a set of unsaturated nutritional compounds that includes retinol, retinal, retinoic acid, and provitamin A carotenoids. It has been found to be involved in many eye functions, such as rod function and dark adaptation [36]. Medical researchers and clinicians believe that normal rod function and dark adaptation are closely related to the synthesis to decomposition ratio of rhodopsin. This process also requires retinal reductase, which needs vitamin A as an activator [37]. Lutein and zeaxanthin are carotenoids found in dark green leafy vegetables and are concentrated around the fovea in the macula in the conical axons. These carotenoids constitute the yellow macular pigment that partially shields the photoreceptors from short-wavelength light, minimizing the effect of chromatic aberration on visual acuity and possibly protecting these cells from oxidative damage [38]. In fact, an increasing amount of evidence suggests that deficiency of vitamin A not only causes night blindness but may also cause structural degeneration of the retina [37, 39]. We found that several studies have shown the protective effect of vitamin A or lutein or docosahexaenoic acid in reducing the risk of RP [10, 40–42]. Garcia-Gonzalez reported a case consistent with cobalamin C deficiency-associated retinopathy [43]. In this condition, the normal conversion of cobalamin (vitamin B12) to its enzymatically active derivatives does not occur, resulting in typical progression to retinal degeneration.

Berson et al. [17] suggested a beneficial effect of 15000 IU/d of vitamin A on the course of RP based on ERG results. Besides, daily intake of a vitamin A supplement may provide protection against possible transient decrease in serum retinol concentration which may adversely affect photoreceptor function. It is worth noting the potential adverse effects of vitamin A supplementation. In fact, excessive vitamin A supplementation was shown to be risky for young RP patients or those with ABCA4 mutations due to the overaccumulation of toxic vitamin A byproducts in the retina [44]. Therefore, patients should consult their ophthalmologist before initiating a potential treatment, although no systemic side effects or toxicity of high-dose vitamin A supplementation or long-term use of vitamin A supplements were found during this systematic review. Pasantes-Morales et al. [18] assessed the effect of taurine/vitamin E formulation on the progression of visual field loss in retinitis pigmentosa. A decrease in visual field loss was observed in the study, likely due to the protection from free-radical reactions in the affected photoreceptors. Moreover, there is substantial evidence that shows the protective effects of taurine on membranes damaged by lipoperoxidation in a variety of cell types [45, 46]. Since all the patients received a combined treatment, the beneficial effects observed could be categorized as synergistic effects. Another study by Berson et al. [19] showed that 1200 mg/d of docosahexaenoic acid supplementation over a 4-year interval did not, on average, slow the course of disease in RP patients. A study by Hoffman et al. [20] suggested that DHA supplemented at a dose of approximately 10 mg/kg/d significantly elevated the red blood cell (RBC)-DHA level, and the cone ERG function was found to be associated with RBC-DHA. Patients with DHA intake greater than 10 mg/kg/d had diminished loss of ERG function. It was also established that the DHA supplementation was beneficial in preserving rod ERG function in patients younger than 12 years and maintaining cone ERG function in patients 12 years and older. Hoffman et al. [24] observed the role of long-term supplementation of DHA on X-linked retinitis pigmentosa and found that DHA supplementation was not effective in slowing the loss of cone or rod ERG function. A lower-than-expected event rate and an underpowered trial due to participant dropout were the primary limitations in the trial. In addition, this trial was limited to X-linked retinitis pigmentosa, and this may be another reason for this result. However, the trial validated the safety of long-term use of DHA supplements. Adults with RP receiving DHA at a dosage of 1200 mg/d for 4 years displayed no adverse effects [19]. Furthermore, Bahrami et al. [21] suggested that lutein supplementation improves the visual field along with a slight improvement in visual acuity. However, the effect of lutein starts several weeks after the supplement intake and persists until weeks after stopping the treatment. Berson et al. [22] showed a positive effect of 12 mg/d lutein supplementation: the mean rate of sensitivity loss in the HFA 60-4 program was lower in the lutein plus vitamin A group than in control tablet plus vitamin A. This study also supported the use of 12 mg/d of lutein to slow visual field loss in the nonsmoking adults with RP taking vitamin A. Similarly, Rotenstreich et al. [23] demonstrated that 300 mg of 9-cis *β*-carotene significantly increased retinal function in RP patients.

We determined that all the eight trials had a low risk of bias for the domains assessed. However, we were unable to extract the result data specified in our protocol from the results described in the trials. The trials appear to have been well designed and conducted. All trials described how to generate random sequences, set blinding, and ensure data integrity. However, some trials concluded from the data that supplemental vitamin A or DHA or lutein or *β*-carotene slows the progression of RP based on the ERG measurements rather than visual field [17, 20, 24]. Only three trials described the genetic profile [17, 19, 22], while others did not define the genetic profile of the participants [18, 20, 21, 23, 24]. This might be a limitation of these trials. However, genetic homogeneity may be the strength for two of the trials [20, 24]. All the trials were based at a single study site, which also reduces genetic heterogeneity. Some of the included trials conducted unplanned subgroup analysis that suggested differential effects of the supplements [19, 20]. The lack of a significant benefit on the primary outcome measure in the analysis may be attributed to the limitations of the study including small sample size and confounding variables such as patient age, compliance, and short follow-up duration.

Due to the limitations of the existing randomized clinical trials, it is difficult to judge the universality of the results, but we believe that they may be suitable for most patients with RP with some clinical symptoms. In a broader sense, these findings suggest that the design and reporting of future trials on patients with RP should consider more vitamins or mineral supplements and other outcome measures from the trials included in this review. In lieu of more radical therapeutic methods, including gene and stem cell therapies, vitamins or minerals might be helpful for RP patients or might also act as an adjuvant in future gene therapy.

## Figures and Tables

**Table 1:** Characteristics of the included studies.

Study	Methods	Participants	Genetic profile	Interventions	Length of follow-up	Outcomes	Adverse events	Notes
Berson et al. [17]	RCT	601 (572) RP participants Age: 18–49 years 62% men and 38% women	Autosomal dominant; autosomal recessive; X-linked; isolate; others	Group A: vitamin A 15000 IU/d plus vitamin E 3 IU/d Trace group: vitamin A 75 IU/d plus vitamin E 3 IU/d Group A + E: vitamin A 15000 IU/d plus vitamin E 400 IU/d Group E: vitamin A 75 IU/d plus vitamin E 400 IU/d	4 years	Primary outcome: cone ERG Secondary outcomes: visual field; visual acuity Losses to follow-up: 29/601 (5%)	1 patient in group A + E resulted in a worsening condition	Subgroup analyses: post hoc analysis conducted in cohort of participants with cone ERG > 0.68 *µ*V (354 participants)
Pasantes-Morales et al. [18]	RCT	72 (62) RP participants Age: 10–50 years 29 men/33 women	Not mentioned	Experimental group: diltiazem 30 mg and vitamin E 400 mg Placebo group: taurine, 1 g	3 years	Primary outcome: visual field Secondary outcomes: full-field ERG; visual acuity Losses to follow-up: 12% of the total	None reported	—
Berson et al. [19]	RCT	221 (208) RP participants Age: 18–55 years 51% men and 49% women	Autosomal dominant; autosomal recessive; X-linked; isolate; others	DHA + A group: 1200 mg/d DHA + 15000 IU/d vitamin A Control + A group: 15000 IU/d vitamin A	4 years	Primary outcome: visual field Secondary outcomes: ERG; visual acuity Losses to follow-up: 13/221 (5%)	None reported	Subgroup analyses: participants not taking vitamin A prior to enrollment and those taking vitamin A for 2 years prior to enrollment
Hoffman et al. [20]	RCT	44 (41) RP participants Age: 4–38 years men only	X-linked	DHA group = 400 mg/d placebo group = corn/soy oil triglyceride	4 years	Primary outcome: ERG secondary outcomes: visual field; visual acuity Losses to follow-up: 3/44 (6%) in total	None reported	Rod ERG in X-linked RP children < 12 years and children ≥ 12 years
Bahrami et al. [21]	RCT	45 (34) RP participants Age (mean ± SD):34 (49.2 ± 9.0) 38% men and 62% women	Not mentioned	Lutein capsules group: lutein capsules 10 mg/day for 12 weeks, 30 mg/day for 12 weeks Placebo group: placebo capsules 24 weeks	24 weeks	Primary outcome: visual acuity Secondary outcomes: visual field Losses to follow-up: 11/45 (24%) lutein capsules group	1 participant on lutein and 2 on placebo had impaired liver function tests at one of their 6-week visits; 2 participants had significant intolerance to multivitamin at the start of the study	—
Berson et al. [22]	RCT	240 (225) RP participants Age (mean ± SD): control group: 40 (1.0); lutein + vitamin A group: 38 (1.00) control group: men 53%; lutein + vitamin A group: men 47%	Autosomal dominant; autosomal recessive; X-linked; isolate; others	Lutein + vitamin A group: 12 mg/d Control tablet + vitamin A group: cornstarch control	4 years	Primary outcome: visual field Secondary outcomes: ERG; visual acuity Losses to follow-up: 15/240 (6%)	1 patient in the control plus vitamin A group died in a motorcycle accident; 2 patients in the lutein plus vitamin A group showed slight elevations of serum liver function levels at year 4	—
Rotenstreich et al. [23]	RCT	34 (29) RP participants Age (mean ± SD): 46.7 (16.9) years 72% men and 28% women	Not mentioned	9-cis *β*-carotene group: capsules containing 300 mg of 9-cis *β*-carotene-rich alga *Dunaliella bardawil* (*β*-carotene, approximately 20 mg) Placebo group: starch 300 mg	A 90-day	Primary outcome: the dark-adapted ERG maximal b-wave response amplitude Secondary outcomes: the light-adapted ERG b-wave response amplitudes visual field; visual acuity Losses to follow-up: 5/34 (14%)	None reported	—
Hoffman et al. [24]	RCT	78 (60) RP participants Age (mean ± SD): DHA group: 16.1 (1.4), placebo group: 14.9 (1.1) men only	X-linked	DHA group = algal-derived DHA (30 mg/kg/d) Placebo group = corn/soy oil triglyceride	4 years	Primary outcome: cone ERG Secondary outcomes: rod ERG Losses to follow-up: 18/78 (23%)	1 participant with a family history of Crohn disease who was sensitive to DHA supplementation; no severe TEAEs	—

**Table 2:** Characteristics of the excluded studies.

Study	Reason for exclusion
Sibulesky et al. [7]	Secondary analysis assessing the safety of long-term vitamin A supplementation in an RCT for RP [9, 17]. No fundus index.
Zorge et al. [25]	Not randomized. A cohort of RP patients and patients with related retinal degenerations followed prospectively before and after administration of lutein. No control group.
Aleman et al. [26]	Not randomized. Patients with the diagnosis of RP or Usher syndrome and normal subjects; a subset of 23 patients with retinal degeneration and 8 normal subjects participated in a 6-month pilot trial of lutein supplementation. No placebo control group and no attempt to mask the patient as to the content of the supplement.
Wheaton et al. [27]	Secondary analysis assessing the safety of long-term DHA supplementation in an RCT for X-linked RP. No fundus index.
Berson et al. [28]	Subgroups of patients with retinitis pigmentosa between rates of decline in ocular function, dietary *ω*-3 fatty acid intake and RBC PE DHA levels and whether vitamin A intake was associated with a change in RBC PE DHA levels over the duration of the study [19].
Rotenstreich et al. [29]	Nonrandomized prospective pilot study, treatment of a retinal dystrophy, fundus albipunctatus, with oral 9-cis-{beta}-carotene. No control group.
Berson et al. [8]	Not randomized clinical trials, calculated dietary intake from 3 clinical trials conducted among patients with typical retinitis pigmentosa from 1984 to 1991 (clinical trial 1), 1996 to 2001 (clinical trial 2), and 2003 to 2008 (clinical trial 3) by questionnaires.
Rayapudi et al. [30]	Not randomized clinical trials, a review.
Hughbanks-Wh eaton et al. [31]	Safety assessment of high-dose docosahexaenoic acid (DHA) supplementation in a 4-year randomized clinical trial in X-linked retinitis pigmentosa (XLRP). No fundus index.
Hoffman et al. [32]	Ancillary outcomes of the DHAX Trial [24].
Berson et al. [33]	No randomized control design. Retrospective, nonrandomized comparison retrospective, observational design of vitamin A, and control cohorts followed up for a mean of 4 to 5 years.
Clinical trials [34]	The effect of oral administration of 9-cis *β*-carotene-rich powder of the alga *Dunaliella bardawil* on visual functions in patients with RP. Recruiting.
Clinical trials [35]	The effect of oral administration of 9-cis *β*-carotene-rich powder of the alga *Dunaliella bardawil* on visual functions in adolescent patients with RP. Not yet recruiting.

*Note.* DHA: docosahexaenoic acid; RCT: randomized controlled trial; RP: retinitis pigmentosa.
